# Open-Label Phase II Study of Olokizumab in Adolescent Patients with Polyarticular Juvenile Idiopathic Arthritis: Results of the 24-Week Treatment Period

**DOI:** 10.3390/ph19010079

**Published:** 2025-12-30

**Authors:** Ekaterina I. Alexeeva, Tatiana M. Dvoryakovskaya, Irina P. Nikishina, Elena S. Zholobova, Valeriya G. Matkava, Elizaveta A. Krekhova, Rinat K. Raupov, Daria V. Bukhanova, Alina N. Egorova, Sergey A. Grishin, Mikhail Yu. Samsonov, Mikhail M. Kostik

**Affiliations:** 1 Department of Pediatric Rheumatology, National Medical Research Center of Children’s Health, Moscow 119991, Russia; alekatya@yandex.ru (E.I.A.); tbzarova@mail.ru (T.M.D.); lizakrek@mail.ru (E.A.K.); 2 Clinical Institute of Children’s Health Named After N.F. Filatov, I.M. Sechenov First Moscow State Medical University (Sechenov University), Moscow 119435, Russia; zholobova_1959@mail.ru; 3 V.A. Nasonova Research Institute of Rheumatology, Moscow 115522, Russia; irpetnik@yandex.ru (I.P.N.); valeriiamatkava@gmail.com (V.G.M.); 4 Pediatric Rheumatology Department, H. Turner National Medical Research Center for Children’s Orthopedics and Trauma Surgery, Saint Petersburg 197136, Russia; rinatraup94@gmail.com; 5 Medical Department, R-Pharm JSC, Moscow 119421, Russia; bukhanova@rpharm.ru (D.V.B.); an.egorova@rpharm.ru (A.N.E.); sa.grighin@rpharm.ru (S.A.G.); samsonov@r-pharm.com (M.Y.S.); 6 Department of Hospital Pediatrics, Saint-Petersburg State Pediatric Medical University, Saint Petersburg 194100, Russia

**Keywords:** juvenile idiopathic arthritis, biologics, bDMARDs, interleukin-6 inhibitor, olokizumab, clinical trial

## Abstract

**Background/Objectives**: This study aimed to evaluate the pharmacokinetics (PK), effectiveness, and safety of the direct interleukin-6 (IL-6) inhibitor olokizumab (OKZ) in adolescent patients with active polyarticular juvenile idiopathic arthritis (pJIA) who had an inadequate response or intolerance to methotrexate (MTX). **Methods**: We analyzed results from an open-label, single-arm trial of OKZ therapy at a dose of 64 mg every 4 weeks for 24 weeks. We evaluated pharmacokinetic (PK) parameters, clinical effectiveness, serum C-reactive protein (CRP) dynamics, and adverse events (AEs). **Results**: Sixteen patients were included in the study, of whom 13 (81.2%) received OKZ through Week 24. The PK profile was consistent with observations in adults with rheumatoid arthritis (RA). By Week 16, 12 (80%) patients achieved an ACRpedi30 response, 11 (73.3%) achieved an ACRpedi50 response, and 2 (13.3%) reached inactive disease status. This response was sustained through Week 24, and no disease flares were observed. A trend toward a better response was noted among patients with baseline CRP > 10 mg/L, higher baseline IL-6, and those naïve to biologic DMARDs. Twelve patients (75.0%) experienced twenty-three mild or moderate AEs. Infections were the most frequent AEs (in 6 patients, 37.5%). No serious AEs or deaths occurred. **Conclusions**: OKZ treatment reduced pJIA disease activity and was well tolerated. The safety profile was consistent with that of other IL-6 inhibitors, and the PK profile matched that seen in adult RA patients.

## 1. Introduction

Juvenile idiopathic arthritis (JIA) is the most common chronic rheumatic disease in children, and its treatment remains challenging. A significant proportion (up to 35%) of all JIA cases fall into the polyarticular (pJIA) category [1]. Patients with pJIA are at high risk of progressive joint involvement, functional impairment, reduced quality of life, disability, and growth retardation [1,2,3,4,5]. More than a quarter of patients with pJIA experience functional limitations within five years after disease onset, and two-thirds showed significant progression in radiographic stage before biologic disease-modifying antirheumatic drugs (bDMARDs) were introduced in pediatric rheumatology [6]. Currently, targeted therapies, including inhibitors of tumor necrosis factor-α (TNF-α), cytotoxic T-lymphocyte-associated protein 4, Janus kinases, interleukin-6 (IL-6), and CD20+ lymphocyte blockers are available; however, choosing the most effective therapy remains challenging.

At least 30% of patients with pJIA continue to have active arthritis despite treatment with methotrexate (MTX) and/or approved bDMARDs, and require new therapeutic options [7]. Interleukin-6 (IL-6), a pro-inflammatory cytokine, is elevated in both blood and synovial fluid, and its level correlates with the severity of joint involvement, functional status, and other inflammatory markers such as C-reactive protein (CRP) and erythrocyte sedimentation rate (ESR) [8,9,10]. IL-6 promotes differentiation and activation of inflammatory cells in the synovium, osteoclast activation, and periarticular inflammation, and induces the production of other pro-inflammatory cytokines [11]. Thus, IL-6 remains one of the most attractive targets for the treatment of JIA.

Among IL-6 pathway inhibitors, the IL-6 receptor inhibitor tocilizumab (TCZ) was approved for the treatment of pJIA in children and adolescents. A second IL-6 receptor inhibitor, sarilumab, was approved in the U.S., but only for patients weighing ≥63 kg. Olokizumab (OKZ; ‘Artlegia’, R-Pharm, Russian Federation) is a humanized monoclonal antibody (IgG4-κ) that directly neutralizes IL-6. Its pharmacokinetics, efficacy, and safety have been established in adult RA patients in three international randomized controlled phase III trials: CREDO 1 (NCT02760368) [12], CREDO 2 (NCT02760407) [13], and CREDO 3 (NCT02760433) [14], as well as in the long-term open-label safety study CREDO 4 (NCT03120949) [15], which enrolled 1785 patients who had competed the randomized trials. The phase III CREDO clinical program demonstrated that OKZ is an effective treatment for moderate-to-severe rheumatoid arthritis in patients with inadequate responses to methotrexate or TNF-α inhibitors, showing superiority over placebo in all primary and key secondary endpoints. Furthermore, the treatment effect was sustained in long-term follow-up studies, with a safety profile consistent with other IL-6 inhibitors. Unlike other IL-6 inhibitors used for RA, OKZ directly blocks IL-6 rather than its receptor.

## 2. Results

**Figure A1:**
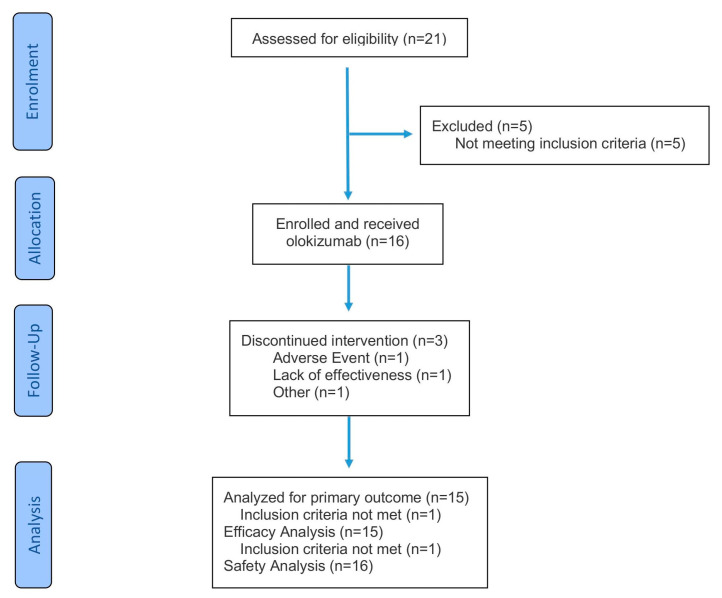
Patient flow diagram.

Following screening, 16 patients initiated OKZ therapy (Appendix A, Figure A1). Of these, 13 (81.2%) received treatment through Week 24. Baseline characteristics are shown in Table 1. Three patients (18.8%) discontinued therapy before Week 24: one was excluded on Day 21 due to misclassification upon diagnostic review (did not meet ILAR pJIA criteria) and was removed from effectiveness and PK analyses; one discontinued due to lack of response; and one discontinued due to an adverse event (described in Section 2.3).

### 2.1. Pharmacokinetics

**Figure 1:**
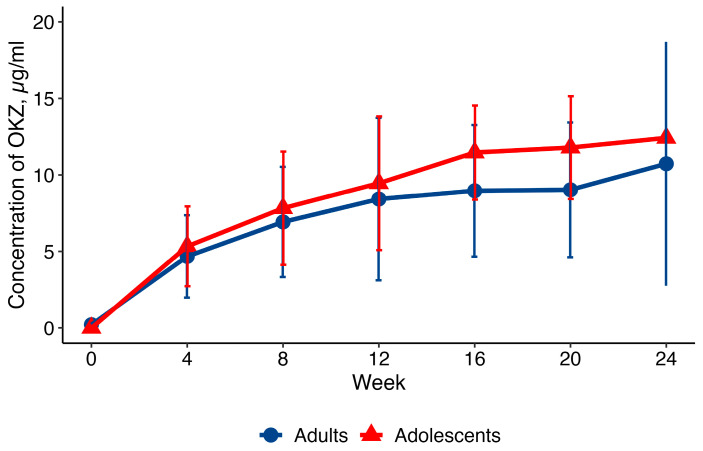
Profile (mean ± SD) of OKZ concentrations (q4w) in adults and adolescents. PK population. SD = standard deviation; q4w = every 4 weeks.

The primary aim was to determine the pharmacokinetic (PK) profile of OKZ and to compare key PK parameters with those in adult RA. After the first dose, the median time to maximum serum concentration (Tmax) was 168 hours (approximately 7 days), and the mean maximum serum concentration (Cmax) was 11.1 ± 4.1 μg/mL. Steady-state concentrations were reached by Week 16, with a mean area under the curve (AUC) of 36,304.5 ± 13,587.9 μg·h/mL. The PK concentration profiles were similar to those in adults from the CREDO 1 trial (Figure 1).

### 2.2. Effectiveness

**Figure 2:**
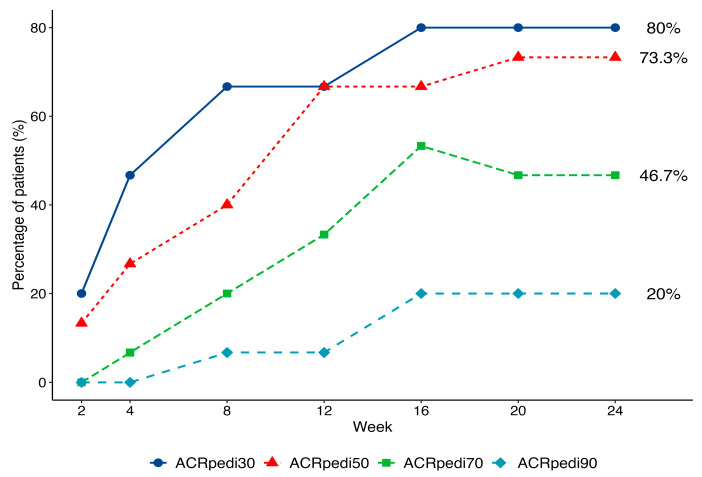
Dynamics of ACRpedi30/50/70/90 response over 24 weeks of treatment.

An ACRpedi response was observed as early as Week 2 and increased over time (Figure 2). By Week 16, 12 of 15 patients (80%) achieved ACRpedi30, 11 of 15 (73.3%) achieved ACRpedi50, 3 of 15 (20%) achieved ACRpedi90, and 2 of 15 patients (13.3%) achieved inactive disease per JADAS criteria. This treatment response was maintained up to the end of treatment (Week 24). By Week 24, 7 of 15 (46.7%) patients had achieved an ACRpedi70 response.

**Figure 3:**
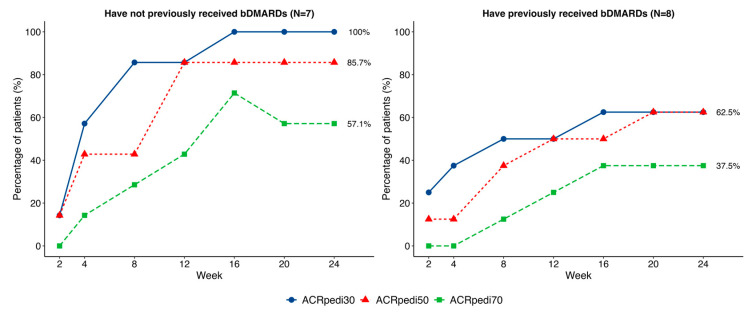
Comparison of ACRpedi30/50/70 responses in patients with and without a history of bDMARDs administration.

Half (50%) of patients had received ≥2 prior bDMARDs (primarily TNF-α inhibitors) because the study did not restrict the number of preceding bDMARDs. Visual but not statistically significant differences in response were noted based on prior bDMARD therapy (Figure 3).

**Figure 4:**
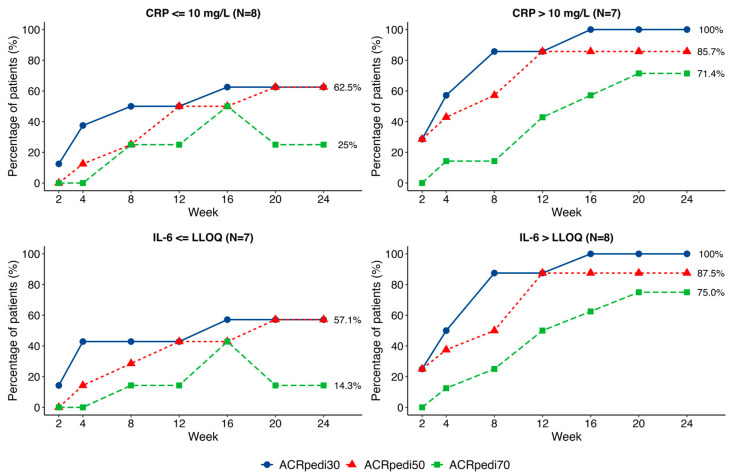
Comparison of ACRpedi30/50/70 responses in patients with different baseline CRP and IL-6 values.

Patients had varied baseline IL-6, ESR, and CRP values. A subgroup analysis suggested a trend toward better ACRpedi responses among those with elevated baseline CRP levels (>10 mg/L). The baseline IL-6 level was >LLOQ (lower limit of quantification) in 8 patients, with a median value of 87.9 (8.0; 106.9) pg/ml, and in 7 patients it was ≤LLOQ. ACRpedi outcomes showed an insignificant trend toward better results in patients with higher baseline IL-6, with a significant difference in ACRpedi70 response (*p* = 0.041; Figure 4).

The Juvenile Arthritis Disease Activity Score 71 (JADAS-71) declined steadily over the 24 weeks. At Week 24, statistically significant reductions (*p* < 0.05) were observed in mean JADAS-71 score (61.5%), number of active joints (66.7%), physician’s global assessment (61.8%), parent’s global well-being score (80.8%), number of joints with functional limitation (50.2%), and ESR (75.0%). The mean Childhood Health Assessment Questionnaire (CHAQ) disability index improved by 66.7% during the trial. CHAQ improvement at Week 24 (−0.43) exceeds the minimal clinically important difference (−0.188). CRP decreased by 81.8% after the first OKZ administration. A summary of these changes is presented in Table 2.

By Week 24, 5 of 15 patients (33.3%) had achieved minimal disease activity according to JADAS criteria. After treatment response was achieved, no pJIA flares occurred, as defined by PRCSG-PRINTO criteria.

### 2.3. Safety

The safety profile of OKZ was consistent with expectations for this drug class, and no new safety signals were identified (Table 3). A total of 12 patients (75.0%) experienced at least one adverse event (AE), all of which were mild or moderate in severity. No Grade 3 or higher AEs, serious adverse events (SAEs), or deaths occurred during the 24-week study.

Infections were the most frequent AEs, reported in 6 (37.5%) patients. All infections resolved without sequelae or complications, and no cases of tuberculosis or opportunistic infections occurred. Only one patient experienced a local injection-site reaction. One case of hepatotoxicity involved an isolated elevation of AST and ALT (less than 2× the upper limit of normal) without clinical symptoms, occurring 7 days after the first dose in a patient concomitantly treated with MTX. Liver enzyme levels normalized within a week.

A subset of patients exhibited transient laboratory abnormalities typical of IL-6 inhibitors. Biochemical analysis revealed transient elevations in AST and ALT levels below twice the upper limit of normal (<2× ULN). Concurrent elevation of both transaminases was documented in two patients one week following OKZ administration, with peak values of 58.9 U/L for AST and 57.8 U/L for ALT (reference ranges: ≤38 U/L and ≤41 U/L, respectively). Bilirubin levels remained within reference ranges in both cases. Both patients were receiving concomitant MTX therapy. No cases meeting the criteria for Hy’s Law were observed.

Isolated instances of decreases from baseline in leukocyte (to 3.04 × 10^9^/L), neutrophil (to 1.13 × 10^9^/L), and platelet (to 174 × 10^9^/L) counts were observed. A non-progressive trend towards decreased leukocyte and neutrophil levels was noted; mean values for these parameters stabilized and remained within the normal range throughout the study. In three patients (18.8%), neutrophil levels decreased below 1.5 × 10^9^/L at individual visits during the 24-week study. These adverse events were neither related to infections nor required treatment or discontinuation of OKZ. Isolated, transient elevations in blood eosinophils were observed in up to 3 patients (18.8%) at weeks 2 and 4, with one instance coinciding with a local injection site reaction.

Throughout the study, sporadic, mild increases in total cholesterol and triglycerides were observed. However, all elevations were mild, and the mean and median values remained within reference ranges, and in some patients, deviations from baseline were noted at the start of the study. Episodes of reduced plasma fibrinogen (down to 1.37 g/L) were documented in up to 4 (30.8%) patients at week 24, but none manifested clinically, and investigators assessed all as clinically insignificant.

A 12-year-old girl with seronegative polyarticular juvenile idiopathic arthritis (pJIA) affecting the ankle, knee, and foot joints, who had previously been treated with etanercept and adalimumab, developed de novo psoriasis at week 12 of treatment, which led to early discontinuation of therapy at the investigator’s discretion.

Overall, OKZ 64 mg administered every 4 weeks was well tolerated in adolescents with pJIA during the 24-week study. The safety profile was consistent with previous data from adult RA studies.

## 3. Discussion

Pediatric rheumatologists currently have access to multiple bDMARDs approved for the treatment of JIA. Nevertheless, the limited effectiveness of available therapies necessitates novel agents targeting diverse biological pathways, among which IL-6 represents a promising therapeutic target. Therefore, OKZ, which directly inhibits IL-6 overproduction, emerges as a potential therapeutic strategy for pJIA.

This study aimed to assess the PK, effectiveness, safety, and tolerability of olokizumab over 24 weeks in adolescent patients with active pJIA who had an inadequate response or were intolerant to MTX.

The study population was balanced for sex and included adolescents of all ages with polyarthritis subtypes as defined by the inclusion criteria. Heterogeneity in prior treatment exposure was observed: half of the patients had received only MTX or MTX plus one bDMARD. At the same time, the remainder had previously failed ≥2 bDMARDs, making OKZ a fourth-line therapy option. The absence of limits on the number of prior bDMARDs suggests a more refractory phenotype, providing a valuable context for interpreting response rates. Baseline JADAS71 scores indicated high disease activity in the studied cohort [17,18].

The PK profile of OKZ, including time to maximum concentration, steady-state achievement, and pre-dose trough concentrations, proved consistent with data from adult RA patients. The overlapping PK curves with adult RA patients (Figure 1) suggest no clinically relevant differences in exposure. Also, the PK profile, i.e., time to C_max_ and time to steady-state, is consistent with IgG4 monoclonal antibody kinetics.

The ACRpedi response criteria and standard efficacy outcomes in pJIA trials [19] were the primary effectiveness measures in this clinical trial. Our results demonstrate the potential therapeutic benefit of OKZ in pJIA patients. Notably, in biologic-naïve patients (N = 7), 100% achieved ACRpedi30 and 85.7% achieved ACRpedi50, while responses were lower in those with prior bDMARDs (N = 8), consistent with earlier reports [20].

Throughout the treatment period, a progressive reduction in JIA disease activity was observed, as evidenced by declining JADAS-71 scores. Changes in both the composite index and its individual components were statistically significant compared with baseline (*p* < 0.05). At week 24, the mean absolute improvement in CHAQ was −0.43, exceeding the threshold for clinically meaningful improvement (−0.188) [21].

The response rates (ACRpedi30 73.3%, inactive disease per JADAS 13.3%) and JADAS71 improvements were consistent with real-world data for TCZ-treated pJIA patients for ≥3 months, where ACRpedi30 was achieved in 70.5–80% of patients and inactive disease in 10.3–16.8% at 6 months, with mean JADAS71 decreasing from 26.5 to 14.0 over this period [22,23,24].

Given cohort heterogeneity, a subanalysis stratified by baseline CRP and IL-6 levels was performed. Patients with elevated baseline CRP (>ULN) and IL-6 (>LLOQ) showed a trend toward more pronounced treatment response—a finding potentially attributable to higher IL-6 activity and greater pathogenic significance of this cytokine in these patients. This pattern mirrors observations with other IL-6 inhibitors in adult RA populations [25] and may reflect an IL-6-driven JIA phenotype. However, these results are exploratory due to the small sample size.

The safety assessment demonstrated favorable tolerability of OKZ in adolescents with pJIA, with a safety profile consistent with that observed in adult RA populations. The most frequent AEs were infections, which align with the immunosuppressive mechanism of OKZ, concomitant MTX therapy, and underlying disease activity [26]. No serious infections occurred during the observation period; all reported infections were mild to moderate in severity and resolved without sequelae. Laboratory abnormalities characteristic of IL-6 inhibition class effects were observed, including mild transaminase elevations (AST/ALT ≤ 2× ULN), reductions in neutrophil counts, and decreased plasma fibrinogen levels. No instances of transaminase elevation met Hy’s Law criteria [27].

Only one AE led to early treatment discontinuation: new-onset psoriasis in a 12-year-old girl with seronegative JIA affecting lower extremity joints. Given the recognized heterogeneity of extra-articular manifestations in pJIA, particularly the frequent delayed onset of psoriatic features in pediatric versus adult populations, as well as previous TNF-α inhibitor exposure, this case likely represents a temporal association rather than drug-induced pathology [28]. No new safety signals emerged during the 24-week treatment period.

This study had several limitations, including its open-label, non-randomized, and uncontrolled design, with no comparator group, which makes it challenging to distinguish the treatment effect from natural fluctuations of disease activity. The absence of restrictions on prior bDMARDs may have influenced the study’s results. Furthermore, it included only patients with a body weight ≥45 kg, limiting generalizability to lower-weight adolescents. Finally, the population was heterogeneous with respect to JIA subtype, prior treatment, and disease duration.

## 4. Materials and Methods

A multicenter, open-label, non-randomized, uncontrolled study CL04041182 evaluated the effectiveness, safety, and PK over 24 weeks of treatment, with an extended analysis planned for week 72. All patients received OKZ 64 mg subcutaneously every 4 weeks. After obtaining written informed consent from both legal guardians and patients, participants entered the screening phase and, upon meeting all eligibility criteria, were enrolled in the treatment phase. During the first month, patients visited the study center weekly; thereafter, visits were monthly. Each visit included assessments of effectiveness and safety, as well as the collection of biological samples for PK analysis. An independent data monitoring committee, comprising pediatric and rheumatology experts, continuously reviewed safety data.

### 4.1. Eligibility Criteria

The study enrolled adolescents aged 12–17 years, with a body weight of at least 45 kg, and active polyarthritis. Eligible patients had a confirmed diagnosis of seropositive or seronegative pJIA, extended oligoarticular JIA, or sJIA with persistent arthritis in the absence of active systemic symptoms, based on ILAR JIA criteria [16], with disease onset before the age of 16. Inclusion criteria also required at least five active joints at screening and baseline, as well as a documented history of MTX intolerance or ineffectiveness. These JIA subtypes share a dominant IL-6-driven inflammatory pathway, making them a rational target for IL-6 blockade to assess efficacy in active polyarticular disease. Inclusion criteria also required at least five active joints at screening and baseline, consistent with the EMA Guideline on the clinical investigation of medicinal products for juvenile idiopathic arthritis (2015). Patients were allowed to continue stable oral glucocorticoids at doses <0.2 mg/kg/day. Prior treatment with JAKi or IL-6 inhibitors was not allowed. There were no limits on the number of prior bDMARDs. The main exclusion criteria comprised recent or severe infections, malignancy, active or latent tuberculosis, hepatitis B or C infection, diverticulitis, primary immunodeficiency syndromes, and laboratory abnormalities at screening, including elevated ALT/AST levels, neutropenia, leukopenia, and thrombocytopenia.

### 4.2. Treatment

OKZ was administered subcutaneously at a dose of 64 mg every 4 weeks. The dose was selected using mathematical modeling to ensure that the expected concentration-time profile in adolescents with body weight ≥ 45 kg matched that observed in adults with RA receiving the same dosing regimen.

### 4.3. Study Endpoints

The primary endpoints were the PK parameters of OKZ following the initial administration and subsequent doses. Blood samples for PK analysis were collected before each injection and on days 3, 7, 14, and 21 after the first dose. The PK method is based on the interaction of olokizumab with goat antibodies to human IL-6 immobilized on the plate surface. The method was validated over a concentration range of 0.5–7 μg/mL.

Effectiveness endpoints included:

1. The Proportion of Patients Achieving ACRpedi30/50/70/90 Response
  *ACRPedi30/50/70/90 is defined as at least 30/50/70/90% improvement from baseline in 3 of 6 variables in the core set, while no more than 1 of the remaining variables can worsen by >30%. The variables in the core set include: physician’s global assessment of disease activity; parent/patient global assessment of overall well-being; functional ability (CHAQ); active joint count; number of joints with limited range of motion; and ESR.*
2. Change in the JADAS-71 [29]
  *The JADAS-71 includes the physician’s global assessment of disease activity; parent/patient global assessment of overall well-being; ESR, normalized to a 0 to 10 scale; and active joint counts.*
3. Proportion of Patients Achieving Minimal or Inactive Disease per JADAS Criteria
4. Changes in Inflammatory Markers (CRP, ESR)
5. Number of Joints with Active Arthritis
6. Physicians’ and Parents’ Global Assessments of Disease Activity and Overall Well-Being
7. Functional Status Using the CHAQ [30]

The associations between the baseline CRP and IL-6 values and the effectiveness outcomes were assessed. IL-6 was detected in blood with the non-commercial ELISA assay (LLOQ = 2 pg/mL).

Safety endpoints included the incidence, nature, and severity of AEs and SAEs; changes in laboratory parameters; vital signs; physical exam findings; ECG results; and chest radiographs or CT scans.

### 4.4. Statistics

Sample size was determined using PK population variability parameters from adult RA patients, aiming for 80% power to ensure the 95% confidence interval for the geometric mean of key PK parameters would lie within the 60–140% equivalence range [31].

PK parameters were evaluated using standard non-compartmental methods based on plasma OKZ concentrations, and the results were summarized descriptively at each time point. These concentrations were graphically compared with data from the CREDO 1 phase III study in adults (n = 64).

Effectiveness outcomes were presented as medians with first and third quartiles (median [Q1; Q3]) to reduce bias due to small sample size and non-normal distributions. Categorical outcomes were shown as absolute and relative frequencies. AEs were classified by system organ class and preferred terms using the MedDRA dictionary version 25.1.

Group comparisons of continuous variables were performed using the Mann–Whitney U-test. Within-group changes from baseline were assessed using the Wilcoxon signed-rank test. Missing post-baseline efficacy data following permanent treatment discontinuation were imputed using the last observation carried forward (LOCF) method under a while-on-treatment analysis framework. No adjustment for multiple comparisons was applied. All statistical tests were two-sided with a significance level of *p* < 0.05. The statistical analyses were performed using R version 4.3.3 (R Foundation for Statistical Computing, Vienna, Austria).

## 5. Conclusions

The treatment of adolescents with active pJIA with OKZ was effective, well-tolerated, and safe. Some patients showed improvements as early as Week 2, with sustained benefits observed over 24 weeks. The safety and PK profiles were consistent with those observed in adults with rheumatoid arthritis and in pediatric patients treated with other interleukin-6 receptor antagonists. The following studies with younger participants and larger sample sizes are ongoing now.

## Figures and Tables

**Table 1:** Baseline Characteristics of Patients.

Characteristic		Olokizumab (n = 16)
Age, years	mean (± SD)	14.4 ± 1.86
	median (Q1; Q3)	14.0 (13.0; 16.5)
	min–max	12–17
Sex	Female, n (%)	9 (56.2%)
	Male, n (%)	7 (43.8%)
Body weight, kg	mean (±SD)	53.8 ± 9.48
	median (Q1; Q3)	51.9 (46.3; 58.5)
	min–max	45.0–79.0
Disease duration, years	mean (±SD)	4.7 ± 4.66
	median (Q1; Q3)	3.4 (0.4; 8.9)
	min–max	0–14
JIA subtype (ILAR 2004) [16]	Systemic *	1 (6.3%)
	Oligoarticular extended	3 (18.8%)
	Polyarticular RF-negative	9 (56.3%)
	Polyarticular RF-positive	3 (18.8%)
Patients on MTX at baseline, n (%)		9 (56.3%)
Patients on oral glucocorticoids at baseline, n (%)		3 (18.8%)
Patients with prior bDMARDs, n (%)		9 (56.3%)
Patients with ≥2 prior bDMARDs, n (%)		8 (50.0%)

* Patient had no systemic features at baseline; systemic features were present at the onset with the following polyarthritis course. Abbreviations: Q1 = first quartile; Q3 = third quartile; SD = standard deviation. Note: n = number of patients; % = percentage of patients calculated relative to the number of patients in the safety population (N). Age (years) is defined as (Visit Date − Date of Birth)/365.

**Table 2:** Changes in effectiveness parameters over 24 weeks of OKZ treatment.

Parameter	Baseline	Week 24	*p*-Value
Active joints, median (Q1; Q3) mean (±SD) min-max	8 (6.5; 17.5) 15.7 ± 15.8 6–61	2 (0.5; 10.5) 8.3 ± 11.4 0–35	0.002
Physician’s VAS, cm, median (Q1; Q3) mean (±SD) min-max	6.0 (4.0; 6.8) 5.7 ± 1.8 3.4–8.5	1.7 (1.2; 3.5) 2.3 ± 1.6 0.2–5.5	<0.001
Parents’ VAS, cm, median (Q1; Q3) mean (±SD) min-max	6.1 (4.8; 7.2) 5.8 ± 1.7 2.3–8.2	1.3 (0.9; 4.8) 2.7 ± 2.5 0.0–7.0	0.001
JADAS71, median (Q1; Q3) mean (±SD) min-max	20.8 (18.4; 32.6) 28.4 ± 16.7 14.1–76.1	9.5 (2.7; 20.8) 13.6 ± 13.3 0.30–45.70	<0.001
CHAQ, median (Q1; Q3) mean (± SD) min-max	1.00 (0.69; 1.44) 1.02 ± 0.49 0.38–2.00	0.25 (0.19; 0.75) 0.59 ± 0.64 0.00–1.88	0.043
ESR, mm/h, median (Q1; Q3) mean (± SD) min-max	15.0 (9.5; 33.5) 25.3 ± 24.3 2.0–88.0	5.0 (2.0; 7.0) 5.9 ± 4.7 2.00–15.00	0.004
CRP, mg/L, median (Q1; Q3) mean (± SD) min-max	7.3 (1.3; 24.6) 21.0 ± 29.1 0.4- 82.2	1.0 (0.2; 2.0) 3.8 ± 10.0 0.2–36.8	0.003

Abbreviations: SD = standard deviation; min = minimum; max = maximum; active joints = number of joints with active arthritis; physician’s VAS = physician’s assessment of disease activity; parent’s VAS = parent’s assessment of patient’s overall well-being.

**Table 3:** Adverse events by System Organ Class and Preferred Term.

System Organ Class Preferred Term	Olokizumab N = 16	
	n (%)	Total AEs
**Number of patients with at least one AE**	12 (75.0)	23
**Blood and lymphatic system disorders**	1 (6.2)	2
Leukopenia	1 (6.2)	1
Neutropenia	1 (6.2)	1
**Cardiac disorders**	2 (12.5)	2
Bundle branch block right *	1 (6.2)	1
Sinus arrhythmia	1 (6.2)	1
**General disorders and administration site conditions**	1 (6.2)	1
Injection site hypersensitivity	1 (6.2)	1
**Hepatobiliary disorders**	1 (6.2)	1
Hepatotoxicity	1 (6.2)	1
**Infections and infestations**	6 (37.5)	10
Gastrointestinal viral infection	1 (6.2)	1
Nasopharyngitis	1 (6.2)	1
Otitis media acute	2 (12.5)	2
Paronychia	1 (6.2)	1
Pharyngitis	1 (6.2)	1
Respiratory tract infection	2 (12.5)	2
Rhinitis	1 (6.2)	1
Varicella	1 (6.2)	1
**Investigations**	3 (18.8)	3
Blood alkaline phosphatase increased	1 (6.2)	1
Blood bilirubin increased	1 (6.2)	1
Blood pressure increased **	1 (6.2)	1
**Nervous system disorders**	1 (6.2)	1
Headache	1 (6.2)	1
**Skin and subcutaneous tissue disorders**	2 (12.5)	2
Psoriasis	1 (6.2)	1
Urticaria	1 (6.2)	1
**Vascular disorders**	1 (6.2)	1
Hypertensive crisis **	1 (6.2)	1

Abbreviations: n—number of patients; %—percentage of patients calculated relative to the total number of patients in the population (N). * Incomplete. ** 144/80 mmHg and 160/90 mmHg.

## Data Availability

The data presented in this study are available on request from the corresponding author due to legal reasons.

## Abbreviations

The following abbreviations are used in this manuscript:

AEs	adverse events
bDMARDs	biologic disease-modifying antirheumatic drugs
CHAQ	Childhood Health Assessment Questionnaire
CRP	C-reactive protein
ESR	erythrocyte sedimentation rate
q4w	every 4 weeks
IL-6	interleukin-6
JADAS-71	Juvenile Arthritis Disease Activity Score 71
LLOQ	LLOQ lower limit of quantification
Cmax	maximum serum concentration
MTX	Methotrexate
OKZ	Olokizumab
PK	Pharmacokinetics
pJIA	polyarticular JIA
SD	standard deviation
SAE	serious adverse event
sJIA	systemic JIA
Tmax	time to maximum serum concentration
TCZ	tocilizumab
TNF-α	tumor necrosis factor-α
ULN	upper limit of normal
